# The Emerging Role of Heme Oxygenase and Its Metabolites in the Regulation of Cardiovascular Function

**DOI:** 10.1155/2012/593530

**Published:** 2012-10-21

**Authors:** David E. Stec, Kazunobu Ishikawa, David Sacerdoti, Nader G. Abraham

**Affiliations:** ^1^Department of Physiology and Biophysics, Center for Excellence in Cardiovascular-Renal Research, The University of Mississippi Medical Center, 2500 North State Street, Jackson, MS 39216, USA; ^2^Department of Cardiology, Center for Medical Education and Career Development, Fukushima Medical University, 1 Hikarigaoka, Fukushima, Fukushima City 960-1295, Japan; ^3^Department of Clinical and Experimental Medicine, Clinica Medica 5, University of Padova, Via Giustiniani 2, 35100 Padova, Italy; ^4^Department of Physiology and Pharmacology, The University of Toledo College of Medicine, Toledo, OH 43614, USA

Heme oxygenase (HO) is an essential enzyme for normal homeostasis of the cardiovascular system. It is the enzyme responsible for the breakdown of heme to iron, biliverdin, and carbon monoxide (CO) gas. Biliverdin is then subsequently reduced to bilirubin by the ubiquitous enzyme biliverdin reductase. HO enzymes are essential for the turnover of red blood cells as well as of heme containing proteins within the cell. However, in recent years numerous studies have demonstrated an important role for HO and its metabolites, CO, and bilirubin in regulating various physiological processes throughout the cardiovascular system. More importantly, alterations in HO have been identified in numerous pathological conditions, and therapies which induce HO have been found to provide beneficial outcomes in numerous cardiovascular disease processes. This special issue consists of 9 original research articles, 4 review articles, and 1 clinical study; all of which highlight the important role that HO enzymes have in the regulation of the cardiovascular system.

The important role of HO in the regulation of kidney function and blood pressure is highlighted in studies by F. Botros et al., S. Quadri et al., and E. Csongradi et al. HO-1 induction with hemin results in marked increases in renal blood flow and glomerular filtration rate (GFR) which are associated with increases in urine flow and sodium excretion. HO-1 induction with hemin also attenuates the decrease in renal blood flow to acute angiotensin II (Ang II) treatment. The important role of HO-1 to counteract the effects of Ang II is further demonstrated in studies in by E. Csongradi et al. in which blockade of intrarenal HO-1 increases blood pressure in Ang II-dependent hypertension through increases in intrarenal superoxide production. The important antioxidant actions of HO and its metabolites are also made evident in studies by E. George et al. who demonstrate that both HO-1 induction and treatment with the HO metabolites CO and bilirubin attenuate the increase in oxidant production in placental explants in response to hypoxia. HO induction as well as CO and bilirubin also attenuate hypoxia-induced sFlt production which has important implications for pregnancy-induced hypertension or preeclampsia. Studies by T. Kawakami et al. also highlight the important antioxidant role of HO and its metabolites. In these studies, HO-1 induction lowers oxidative stress levels which in turn improves vascular function, lowers blood pressure, and prevents renal injury in extracellular superoxide dismutase (EC-SOD, SOD3) knockout mice. The important role of HO in the modulation of oxidative stress in the differentiation of bone-marrow-derived mesenchymal stem cells (MSCs) and its potential role in diseases such as diabetes, inflammation, osteoporosis, and hypertension are reviewed by L. Vanella et al. HO-1 regulates MSC differentiation process by shifting the balance of MSC differentiation in favor of the osteoblast lineage by decreasing oxidative stress and increasing osteogenic markers such as alkaline phosphatase and BMP-2.

The important role of HO in target organ injury is also highlighted in this special issue. K. Chandrashekar et al. demonstrate the important role of HO-1 in preserving renal function and protecting the kidney against renal injury in a model of subpressor Ang II-induced kidney injury. M. Constantin et al. detail the therapeutic potential of HO and its metabolite CO in lung inflammation, acute lung injury, lung transplantation, and pulmonary hypertension.

HO enzymes play a critical role in the regulation of vascular function and the protection of the vasculature from injury. The important role of HO enzymes in this capacity as well as the potential targeting of vascular HO for therapeutics is reviewed by E. Marcantoni et al. The importance of vascular HO-1 in the suppression of vascular inflammation is demonstrated in studies by K. Ishikawa et al. who show that HO-1 knockout mice exhibit enhanced vascular inflammation, atherosclerotic lesions, and increased oxidation of HDL.

HO enzymes have an important interaction with nitric oxide (NO) in the regulation of vasculature tone. HO in the vasculature has been previously reported to both induce as well as inhibit the formation of NO depending on the degree of HO-1 induction. A. Daiber et al. examine the complex relationship between HO and NO and its implications in organic nitrate therapy and argue for a beneficial role of HO-1 induction to protect against tolerance to effective organic nitrate-based therapies for cardiovascular disease. The relationship between HO and NO is further investigated in a clinical study by D. Sacerdoti et al. who demonstrate the important role for HO in the maintenance of blood flow in the face of NO inhibition as well as the important role for HO in protecting the bioavailability of vascular-derived NO in humans.

The role of HO and its metabolites in metabolic diseases such as diabetes and obesity is also highlighted in this special issue. A. Elmarakby et al. report that induction of HO-1 is able to lower renal oxidative stress, inflammation, and injury in diabetic spontaneously hypertensive rats (SHRs) suggesting that induction of HO-1 may be a potential therapeutic approach for diabetic nephropathy. J. Cao et al. elegantly demonstrate that HO-1 induction with the Apo AI mimetic, L-4F, decreases blood pressure, insulin resistance, blood glucose, and adiposity in HO-2 knockout mice. These results suggest that HO-1 induction could be a viable therapeutic approach for the correction of both metabolic and cardiovascular disorders in obesity.

Taken together, the series of articles in this special issue highlight the important regulatory role of HO and its metabolites in the cardiovascular system. The articles also demonstrate the important role that HO enzymes play in protecting the cardiovascular system from diseases such as hypertension, atherosclerosis, kidney disease, diabetes, and obesity. They also shed light on the potential therapeutic role for HO-1 induction to combat these diseases. It is clear from these articles that the HO system is indeed emerging as both an important regulator of cardiovascular function as well as an attractive target for the development of effective therapeutics.

